# Interrater reliability of interictal EEG waveforms in Lennox–Gastaut Syndrome

**DOI:** 10.1002/epi4.12858

**Published:** 2023-11-28

**Authors:** Derek K. Hu, Mandeep Rana, David J. Adams, Linda Do, Daniel W. Shrey, Shaun A. Hussain, Beth A. Lopour

**Affiliations:** ^1^ Department of Biomedical Engineering University of California Irvine California USA; ^2^ Division of Neurology Children's Hospital Orange County Orange California USA; ^3^ Department of Pediatrics University of California Irvine California USA; ^4^ Division of Pediatric Neurology University of California Los Angeles California USA

**Keywords:** epilepsy, epileptic encephalopathy, generalized paroxysmal fast activity, interictal epileptiform discharge, sleep spindle, spike and slow wave

## Abstract

**Objective:**

Identification of EEG waveforms is critical for diagnosing Lennox–Gastaut Syndrome (LGS) but is complicated by the progressive nature of the disease. Here, we assess the interrater reliability (IRR) among pediatric epileptologists for classifying EEG waveforms associated with LGS.

**Methods:**

A novel automated algorithm was used to objectively identify epochs of EEG with transient high power, which were termed events of interest (EOIs). The algorithm was applied to EEG from 20 LGS subjects and 20 healthy controls during NREM sleep, and 1350 EOIs were identified. Three raters independently reviewed the EOIs within isolated 15‐second EEG segments in a randomized, blinded fashion. For each EOI, the raters assigned a waveform label (spike and slow wave, generalized paroxysmal fast activity, seizure, spindle, vertex, muscle, artifact, nothing, or other) and indicated the perceived subject type (LGS or control).

**Results:**

Labeling of subject type had 85% accuracy across all EOIs and an IRR of κ =0.790, suggesting that brief segments of EEG containing high‐power waveforms can be reliably classified as pathological or normal. Waveform labels were less consistent, with κ =0.558, and the results were highly variable for different categories of waveforms. Label mismatches typically occurred when one reviewer selected “nothing,” suggesting that reviewers had different thresholds for applying named labels.

**Significance:**

Classification of EEG waveforms associated with LGS has weak IRR, due in part to varying thresholds applied during visual review. Computational methods to objectively define EEG biomarkers of LGS may improve IRR and aid clinical decision‐making.

**Key point**

- A 15‐second EEG segment containing at least one high‐power waveform can be reliably classified as pathological or normal.
- Labeling of subject type (epilepsy vs. control) had Cohen's kappa *κ* = 0.790, while labeling individual EEG waveforms had *κ* = 0.558.
- Many mismatches in EEG waveform labels occurred when one rater selected “nothing,” indicating different thresholds between raters.

## 1 INTRODUCTION

Lennox–Gastaut Syndrome (LGS) is a severe, childhood‐onset epileptic encephalopathy that often evolves from earlier epilepsies such as infantile epileptic spasms syndrome (IESS). LGS is characterized by (1) the presence of multiple seizure types, (2) cognitive impairment, and (3) the presence of interictal epileptiform activity in the electroencephalogram (EEG), such as slow spike–wave (<2.5 Hz) and generalized paroxysmal fast activity (GPFA), a unique waveform that is primarily seen in LGS during NREM sleep.^1^ In addition to their importance for diagnosis, these waveforms can also be biomarkers of treatment response in LGS. For example, a decrease in GPFA burden has been associated with similar reductions in diary‐recorded seizures,^2^ and continuous spike and wave pattern during slow wave sleep has been associated with neurodevelopmental disabilities.^3^ Identifying interictal epileptiform activity can also help classify the type of epilepsy and help clinicians manage anticonvulsants.^4^

The prompt diagnosis of LGS is critical for seizure control and maximizing long‐term neurocognitive outcomes.^5^, ^6^, ^7^ However, because the onset of LGS is often insidious, it can be difficult to define a single timepoint at which LGS begins. Experts may disagree as to whether EEG abnormalities are sufficiently severe to substantiate a diagnosis of LGS, which may delay diagnosis and effective treatment. Interpretation of individual EEG waveforms is inherent to this decision, and low interrater reliability (IRR) could be a significant contributing factor. The reliability of identifying interictal epileptiform discharges has been shown to be limited, as individual experts apply different thresholds in their decisions to mark events.^8^ A consensus has also not yet been reached on the defining characteristics of GPFA, as studies using visually marked events report different values of GPFA amplitude, duration, and frequency across subjects.^9^, ^10^ One group developed an automated detector that defined GPFA as a low‐frequency component (0.3–3 Hz) plus a high‐frequency component (8–20 Hz); in comparison to manually marked GPFA, the detector was found to return a high number of false‐positive detections.^11^ This further highlights a lack of characterization of the specific waveform features used in the visual analysis of LGS EEG, which is a barrier to developing objective biomarkers of LGS. Given these uncertainties, the goal of this study was to evaluate the rater accuracy and IRR of automatically detected EEG waveforms in both healthy controls and patients with LGS.

## 2 METHODS

### 2.1 Subject information

Approval for this retrospective study was obtained from the Institutional Review Boards at the Children's Hospital of Orange County (CHOC) and the University of California Los Angeles (UCLA), with the requirement for informed consent waived. Twenty subjects diagnosed with LGS (7 females, median age 7.4 years, age range 1.0–18.8 years) were retrospectively identified using ICD 9 and 10 diagnostic codes from the clinical record at CHOC, with visits and EEG studies performed between January 2012 and June 2020. The electroclinical diagnosis of LGS was confirmed by a board‐certified pediatric epileptologist. The median time from seizure onset to EEG collection in the cohort was 4.2 years and the median time from LGS diagnosis to EEG collection was 0.1 years (Supplementary Table S1). Twenty healthy control subjects (8 females, median age 8.2 years, age range 1.0–17.7 years) were selected from a cohort of fifty subjects collected in a prior study. The twenty healthy subjects were selected such that they were approximately age‐matched to the LGS group. The cohort of fifty control subjects was retrospectively identified from the clinical record at UCLA with visits between February 2014 and July 2018.^12^ Controls were included if they had (1) no known neurological disorders, (2) a normal overnight video‐EEG, (3) EEG events that were not seizures and were deemed neurologically normal by the attending neurologist, and (4) no use of anti‐seizure medications. There were no significant differences in age between control and LGS subjects (*P* = 0.672, Student's *t*‐test); the breakdown by sex was approximately the same between both groups.

### 2.2 EEG acquisition and preprocessing

All EEG data were recorded using the Nihon Kohden EEG acquisition system, with nineteen scalp electrodes placed according to the international 10–20 system (Fp1, Fp2, F3, F4, C3, C4, P3, P4, O1, O2, F7, F8, T3, T4, T5, T6, Fz, Cz, Pz). The data were recorded at a sampling rate of 200 Hz or downsampled to 200 Hz. For each LGS subject, an EEG clip of NREM sleep lasting 10 minutes containing no arousals or artifacts was selected. First, periods of NREM sleep were identified between the times of 12:00–4:00 AM by a pediatric epileptologist (MR). Three subjects were not asleep during this time window; one subject had NREM sleep from 4:00–6:00 AM and two subjects had NREM sleep from 6:00 PM to 12:00 AM the night before. Then, using an automated artifact detection algorithm, the first clean 10‐minute clip of NREM sleep was selected for analysis. For each control EEG recording, a pediatric epileptologist clipped 20–30 minutes of NREM EEG using a randomization method to select the time point, as described in *Smith* et al. *(2021)*.^12^, ^13^ For each subject, one 10‐minute clip of clean, continuous, NREM sleep EEG with no automated or clinically detected artifacts was selected for analysis. All EEG data were re‐referenced to the common average and filtered with a zero‐phase shift digital filter from 0.5–55 Hz. All electronic data were deidentified and analyzed offline using custom MATLAB (Mathworks) scripts.

### 2.3 Identifying EEG events of interest

The EEG data were first prewhitened in the time domain using first‐order backward differencing to counteract the expected 1/f decrease in power.^14^, ^15^, ^16^ The time‐varying power spectrum of the EEG for frequencies from 1 to 50 Hz was subsequently obtained using the Stockwell transform,^17^ in increments of 1 Hz.

We then identified regions of high power in the time‐varying power spectrum that exceeded a threshold of 250, which corresponded to a z‐score of 1.69 for the EEG power and resulted in selection of 4.6% of all time‐frequency values. This relatively low value for the threshold was chosen to ensure that all relevant waveforms, particularly epileptiform activity, would be captured. This fixed threshold was used across all frequency bands and subjects, as the prewhitening step served to normalize the power across frequency bands and between LGS and control subjects.

**FIGURE 1:**
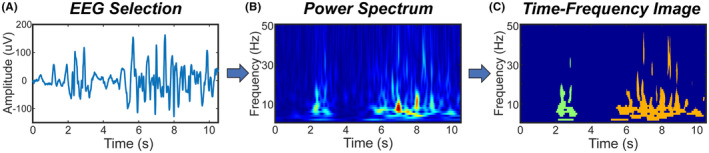
A representative example of EOI identification. (A) 10 minutes of artifact‐free EEG were selected from each subject; a sample of 10 seconds is shown here. (B) The time‐varying power spectrum was calculated for the entire EEG clip after prewhitening. (C) EOIs were identified as consecutive time points in which the EEG power exceeded a threshold. For a single EOI, the set of points in the time‐frequency space that exceeded the threshold was defined as the time‐frequency image (TFI). Here, two different TFIs are shown, one in green and one in orange.

For each EEG, we defined events of interest (EOIs) to be regions of high power in the time‐varying power spectrum that were continuous in time (Figure 1). Specifically, a single EOI consisted of consecutive time points in which the power for at least one frequency exceeded the threshold, with a minimum duration of 100 milliseconds. This minimum length was chosen to fully capture the duration of an epileptic spike, the shortest abnormal waveform we expected to see. Note that epileptic spikes and other sharp waveforms will appear to have a longer duration in the time‐varying power spectrum, such that even a 20 millisecond spike can have a time‐frequency duration exceeding 100 milliseconds (Supplementary Figure S1). EOIs were identified in the EEG data from the Fz electrode, as the frontocentral location should be minimally impacted by eye movements and muscle artifact and maximally sensitive to events such as sleep spindles and GPFAs.^10^

### 2.4 Time‐frequency image features for each EOI

For each EOI, we calculated six features using the time‐varying power spectrum. We first defined the time‐varying power spectrum as $X_{t,f,e}$ where t is time, f is frequency, and e is the electrode at which the EEG was measured (Figure 1B). We then defined a time‐frequency image (TFI) to be the time‐frequency representation of a single EOI. Specifically, the TFI was the set of ordered doublets whose power in an electrode e exceeded the threshold T:

(1) $${\mathrm{TFI}}_{e}=\left\{\left(t,f\right)\;|\;X_{t,f,e}>T\right\}$$

**FIGURE 2:**
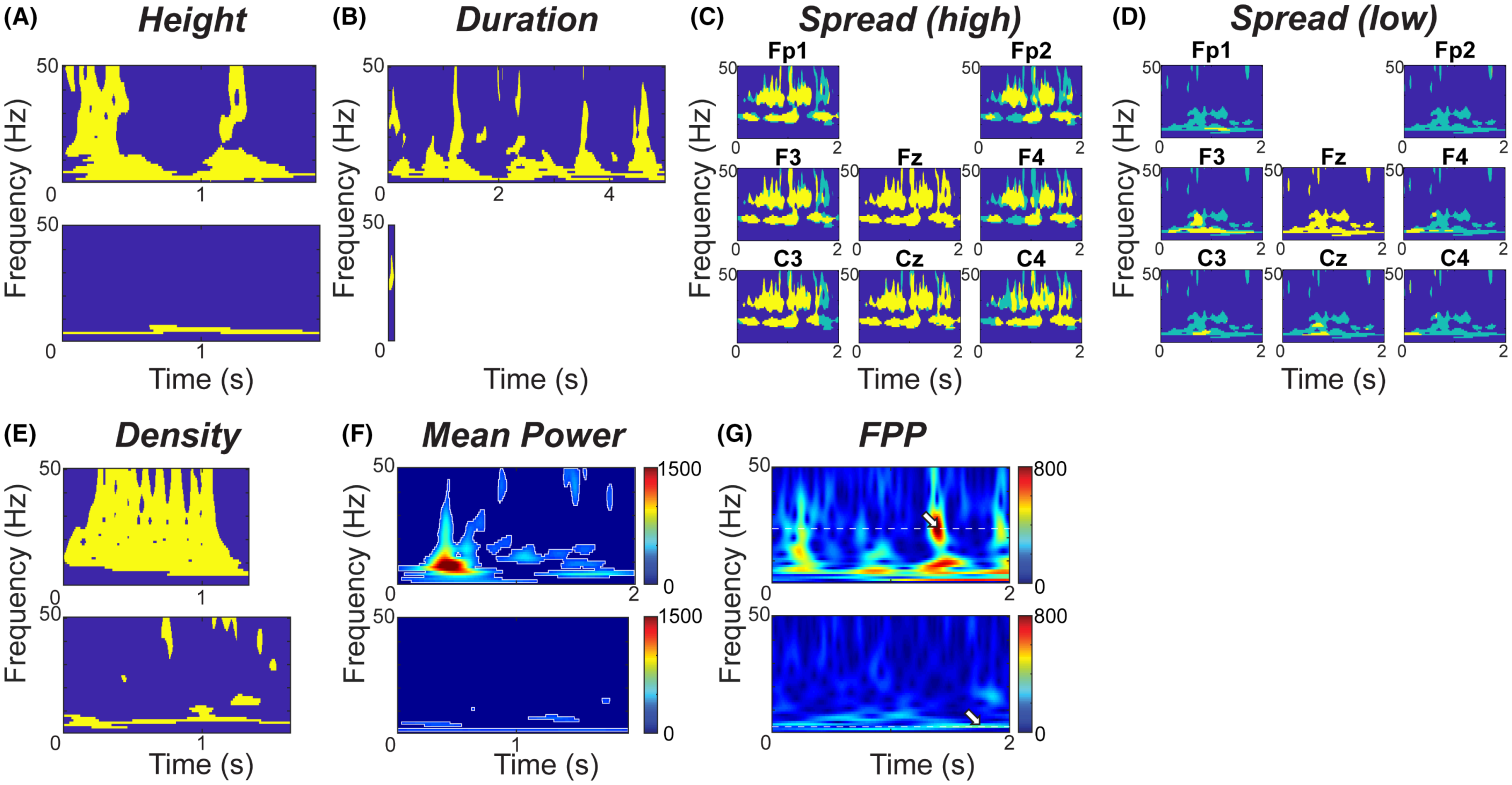
Examples of TFIs and time‐varying power spectrum features: (A) height, (B) duration, (C) high spread, (D) low spread, (E) density, (F) mean power, and (G) frequency of peak power. For all features except spread, an example of a high value is shown at the top of the subfigure, and an example of a low value is shown at the bottom. The spread is visually represented using electrodes adjacent to Fz, with high and low spreads shown in separate subfigures. Yellow regions indicate where the Fz TFI overlaps with the TFIs in adjacent electrodes, while light blue regions indicate where the Fz TFI does not overlap with the TFIs in adjacent electrodes.

Two examples of EOIs and their associated TFIs are shown in Figure 1C. Given these definitions, the six features were as follows:

1. **Height** (Figure 2A): The height was defined by the highest frequency minus the lowest frequency in the TFI for electrode Fz:

(2) $$\text{Height}=\max_{f}\left(\left\{{\mathrm{TFI}}_{\mathrm{Fz}}\right\}\right)-\min_{f}\left(\left\{{\mathrm{TFI}}_{\mathrm{Fz}}\right\}\right)+1$$

The height ranged from 1 to 50 Hz, as the frequencies ranged from 1 Hz to 50 Hz.

2 **Duration** (Figure 2B): EOI duration corresponded to the TFI length in seconds in electrode Fz and was defined by:

(3) $$\text{Duration}=\max_{t}\left(\left\{{\mathrm{TFI}}_{\mathrm{Fz}}\right\}\right)-\min_{t}\left(\left\{{\mathrm{TFI}}_{\mathrm{Fz}}\right\}\right)$$

3 **Spread** (Figure 2C,D): The EOI spread measured how many other electrodes had TFIs that overlapped the Fz TFI in time and frequency. A spread of 1/19 = 0.05 indicated that no other scalp electrodes exhibited high EEG power (exceeding the threshold) at the same time and frequency as the Fz electrode. A spread of 1 indicated that all other electrodes exhibited high EEG power at the same time‐frequency points as the Fz electrode. The spread was defined by:

(4) $$\text{Spread}=\frac{\sum_{e=1}^{\text{nElec}}\left|\left\{{\mathrm{TFI}}_{\mathrm{Fz}}\cap {\mathrm{TFI}}_{e}\right\}\right|}{\text{nElec}*\left|\left\{{\mathrm{TFI}}_{\mathrm{Fz}}\right\}\right|}$$

where $\left|\left\{{\mathrm{TFI}}_{\mathrm{Fz}}\cap {\mathrm{TFI}}_{e}\right\}\right|$ is the cardinality of the intersection between TFI sets for Fz and electrode e, nElec is the total number of EEG electrodes, and $\left|\left\{{\mathrm{TFI}}_{\mathrm{Fz}}\right\}\right|$ is the cardinality of the TFI in Fz.

4 **Density** (Figure 2E): The density was the area of the TFI, normalized by the duration and the maximum possible height (here 50 Hz). A density of 1/50 = 0.02 indicated that the EOI had high power at a single frequency at each time point, and a density of 1 indicated that all possible frequencies exceeded the power threshold at all time points. The density was defined by:

(5) $$\text{Density}=\frac{\left|\left\{{\mathrm{TFI}}_{\mathrm{Fz}}\right\}\right|}{\text{nFreq}*\text{Duration}*\mathrm{fs}}$$

where nFreq is the number of frequencies analyzed and fs is the sampling rate.

5 **Mean Power** (MP, Figure 2F): For each Fz EOI, the mean power was calculated across all time‐frequency points that exceeded the threshold:

(6) $$\mathrm{MP}=\frac{\sum_{\left(t,f\right)\in {\mathrm{TFI}}_{\mathrm{Fz}}}X_{t,f,\mathrm{Fz}}}{\left|\left\{{\mathrm{TFI}}_{\mathrm{Fz}}\right\}\right|}$$

where $\left(t,f\right)$ consists of all ordered doublets within ${\mathrm{TFI}}_{\mathrm{Fz}}$.

6 **Frequency of Peak Power** (FPP, Figure 2G): The FPP was the frequency at which the EOI had the maximum power:

(7) $$\mathrm{FPP}={\text{argmax}}_{f}\left(X_{t,f,\mathrm{Fz}}\right)$$

The FPP ranged from 1 to 50 Hz, based on the frequency range included in the time‐varying power spectrum.

### 2.5 Clustering analysis

Prior to clustering, each feature was normalized by converting the values to a z‐score based on the mean and standard deviation of each feature across all control and LGS EOIs. Control and LGS EOIs were collectively partitioned into twelve different clusters using K‐means.^18^ Each EOI was treated as a single observation; each observation consisted of six variables, which were the z‐scores of the features: height, duration, spread, density, MP, and FPP. A large number of clusters was used to ensure that all combinations of TFI features were represented, thus resulting in a broad distribution of EOIs for visual analysis. For organizational purposes, the clusters were named in descending order based on the sum of the six TFI feature z‐scores, such that cluster one had the highest sum of the six TFI feature z‐scores, and cluster twelve had the lowest sum.

### 2.6 Visual analysis of EOIs

**FIGURE 3:**
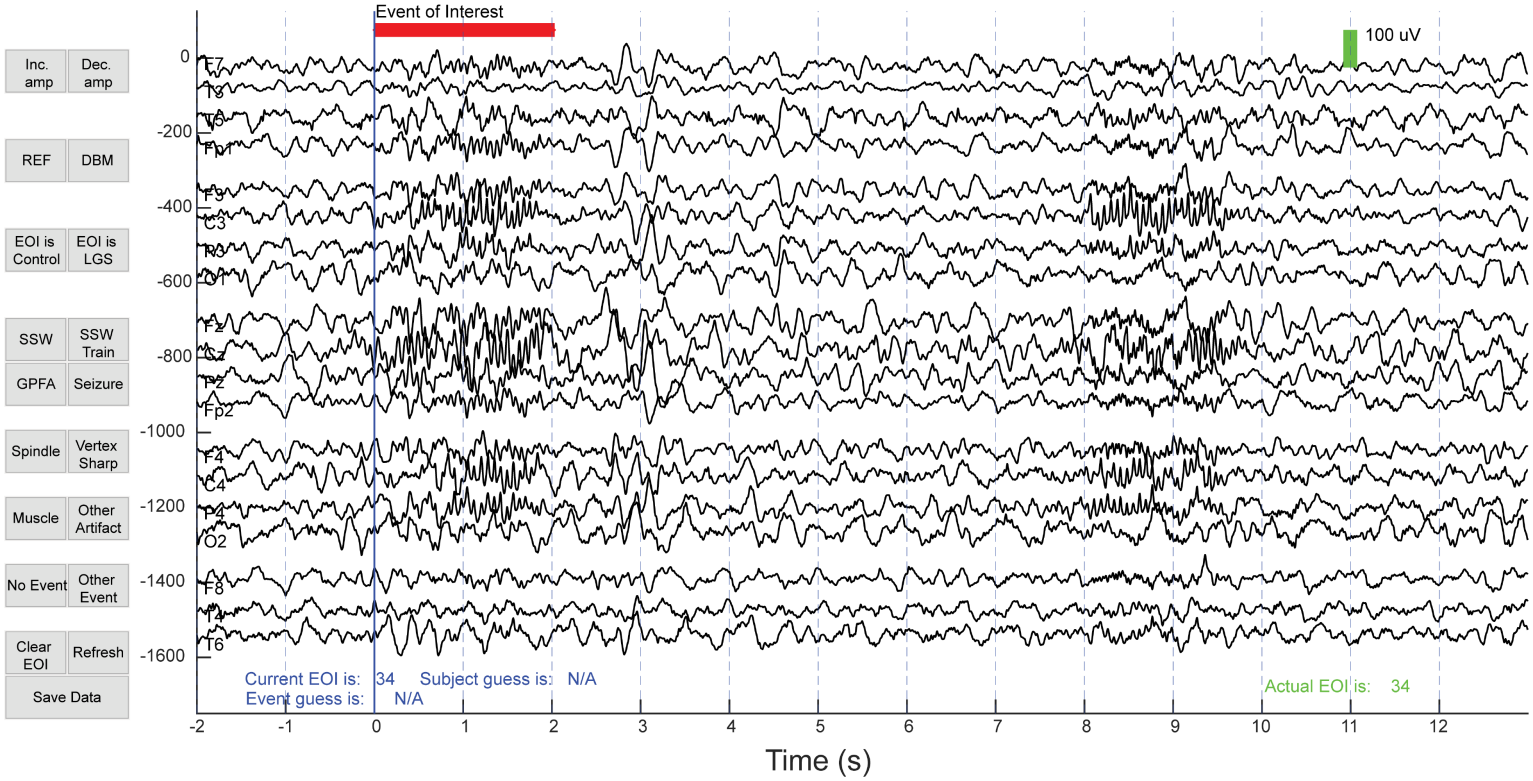
An example of the blinded visual analysis of an EEG EOI. The EOI is indicated by the horizontal red line. For each EOI, the rater selected a subject type and a waveform type. The user interface also included the option to increase and decrease the EEG amplitude scale and change the reference montage between a common average reference montage and a longitudinal bipolar montage.

To ensure the selection of a representative distribution of EOIs for visual classification, 120 EOIs were randomly selected from each cluster (60 from LGS patients and 60 from control subjects). If a cluster contained fewer than sixty control or LGS EOIs, the remaining EOIs were selected from the other cohort. Cluster one consisted of only 30 EOIs, and all EOIs were included in this cluster. In total, 1350 out of 11 708 EOIs were selected for visual analysis. Three board‐certified pediatric epileptologists (DA, DS, SH) from two different institutions (CHOC and UCLA) each classified 900 of the 1350 EOIs, so each EOI was independently classified by two different raters. The order of EOIs was randomized. For each EOI, raters were given a fifteen‐second segment of EEG, starting 10 seconds prior to the EOI and ending 13 seconds after the EOI began, with a red line indicating when the EOI started and ended (Figure 3). Raters were blinded to the subject type, subject number, and the time at which the EEG segment was recorded. For each EOI, raters were asked to determine (1) the subject type, which could be either a control or LGS subject and (2) the waveform type, which could be spike and slow wave (SSW), GPFA, seizure, sleep spindle, vertex sharp, muscle, artifact, other event, or nothing. EOIs were viewed and classified using a custom GUI that was designed using MATLAB in consultation with the clinical team.

### 2.7 Statistical analysis

The IRR reflects the proportion of consistent ratings between clinicians that are not accounted for by chance. IRR was calculated for both subject type and waveform type and was assessed using Cohen's Kappa^19^ and intraclass correlation coefficient (ICC).^20^ Cohen's Kappa ranges from negative one to one, with −1.00‐0.00 = “no agreement”; 0.01–0.20 = “slight”; 0.21–0.40 = “fair”; 0.41–0.60 = “moderate”; 0.61–0.80 = “substantial”; and 0.81–1.00 = “almost perfect or perfect” IRR.^21^ The ICC was calculated using a 2‐way mixed‐effects model, where an ICC greater than 0.7 is generally considered adequate and an ICC greater than 0.9 is considered excellent. The consistency in subject labels and waveform labels between raters was measured using the EOI agreement rate (EAR)^22^:

(8) $$\mathrm{EAR}=\frac{\left|\left\{A\cap B\right\}\right|}{\left|\left\{A\cup B\right\}\right|}$$

where A represents the set of EOIs labeled with a specific subject or waveform type by reviewer A, and B represents the set of EOIs of the same subject or waveform type labeled by reviewer B.

For the subject type, we also determined the accuracy of the visual classification relative to the ground truth of whether the subject had been diagnosed with LGS or not. The accuracy of the subject type labels was calculated as a percentage based on the subject type from which each EEG EOI originated.

## 3 RESULTS

### 3.1 Automated EOI detection identified a broad range of waveform types from all subjects

A total of 11 708 EOIs were detected using the process described in Section 2.3, with 6744 EOIs coming from the twenty control subjects (n_controls_ = 365.0 [259.0–443.0] EOIs per subject; reported as the median [Q1‐Q3] for all results) and 4964 EOIs coming from the twenty LGS subjects (n_LGS_ = 232.5 [156.0–324.5]). The minimum number of EOIs for a single subject was 49, and the maximum number of EOIs was 560. There were no clusters composed of EOIs from a single subject; on average, each subject contributed EOIs to 8.5 different clusters (Supplementary Figure S2). Each cluster was characterized by a different combination of the six features (Supplementary Figure S3). For example, the EOIs in cluster one had a long duration and high spread, while EOIs in cluster two had high density and high mean power. Some clusters had low values of all features; this was expected, as EOIs were identified using a relatively low power threshold to maximize the sensitivity of initial detection. The broad distribution of EOIs across subjects and clusters and the unique characterization of each cluster based on the six features suggest that our algorithm was successful in identifying a broad range of waveforms that were well‐represented in both LGS and control subjects. Clustering EOIs using a power threshold of 200 (7.7% of the data) and 300 (3.0% of the data) produced qualitatively similar clusters (Supplementary Figure S4). For the visual analysis, 60% of the EOIs (n = 813 out of 1350) were from LGs subjects, and 40% (n = 537) were from control subjects.

### 3.2 Classification of subject type was accurate and consistent between raters

The IRR for the determination of subject type (ie, LGS vs. control) associated with each EOI was favorable, with *κ* = 0.790 and ICC = 0.790 (95% confidence interval [CI] 0.769–0.809) across the 1350 EOIs selected for visual analysis (Table 1). Both control and LGS subject labels had similar agreement between raters, with an EAR of 0.806 and 0.813, respectively (Table 2). The mean accuracies for subject classification were 84.4%, 84.7%, and 86.0% for the three reviewers, with significantly lower accuracies for EOIs from LGS subjects compared to EOIs from control subjects for all three raters (Supplementary Table S2; *P* < 0.05, Mann–Whitney U‐test). There were no significant differences in accuracy between the three raters across all LGS and control subjects (*P* > 0.05, Mann–Whitney U‐test; 3 of 3 comparisons).

**TABLE 1:** Interrater reliability for classification of subject type and waveform type for EEG EOIs.

Label type	Rater	Count	Agreement	Kappa	ICC	95% CI
Subject	A‐B	450	0.907	0.813	0.814	0.780–0.843
	A‐C	450	0.916	0.831	0.831	0.798–0.859
	B‐C	450	0.862	0.725	0.725	0.678–0.767
	All	1350	0.895	0.790	0.790	0.769–0.809
Waveform	A‐B	450	0.671	0.560	0.627	0.567–0.681
	A‐C	450	0.682	0.572	0.685	0.633–0.731
	B‐C	450	0.671	0.538	0.697	0.646–0.741
	All	1350	0.675	0.558	0.669	0.638–0.698

*Note*: The three visual reviewers are listed as raters A, B, and C.

**TABLE 2:** EOI agreement rate for subject type and waveform type.

Category		EOI agreement rate (n)			
		A‐B	A‐C	B‐C	All
Subject type	Control	0.831 (248)	0.834 (229)	0.757 (255)	0.806 (732)
	LGS	0.828 (244)	0.853 (259)	0.759 (257)	0.813 (760)
Waveform type	SSW	0.720 (168)	0.727 (154)	0.682 (154)	0.710 (476)
	GPFA	0.400 (25)	0.378 (45)	0.151 (53)	0.285 (123)
	Seizure	0.000 (1)	0.000 (3)	0.000 (0)	0.000 (4)
	Spindle	0.500 (82)	0.480 (100)	0.492 (59)	0.490 (241)
	Vertex	0.100 (40)	0.053 (19)	0.194 (31)	0.122 (90)
	Muscle	0.360 (25)	0.000 (15)	0.000 (18)	0.155 (58)
	Artifact	0.111 (9)	0.167 (12)	0.000 (11)	0.094 (32)
	Nothing	0.561 (205)	0.577 (215)	0.631 (241)	0.592 (661)
	Other	0.023 (43)	0.100 (30)	0.065 (31)	0.058 (104)

*Note*: The three visual reviewers are indicated by A, B, and C. The number in parentheses represents the number of EOIs given that subject or waveform label by either reviewer.

### 3.3 Interrater reliability for waveform type was inadequate, with some very low agreement rates

The IRR for waveform type had κ values ranging from 0.538 to 0.572, with a mean value of 0.558 (Table 1). The ICC between raters was also below adequate, with an ICC of 0.669 (CI 0.638–0.698). The highest number of consistent ratings between reviewers were for EOIs labeled as SSWs (n = 338, EAR = 0.710), nothing (n = 391, EAR = 0.592), and sleep spindles (n = 118, EAR = 0.490), although the EAR values for nothing and sleep spindles did not indicate high levels of agreement. The high number of EOIs labeled “nothing” further confirms the highly sensitive nature of the automated algorithm for identification of waveforms. The EAR values for other waveforms, such as GPFAs (n = 35 with matching labels, EAR = 0.285) and vertex waves (n = 11, EAR = 0.122), were low, indicating significant disagreement in identifying LGS waveforms and sleep architecture, respectively (Table 2).

**FIGURE 4:**
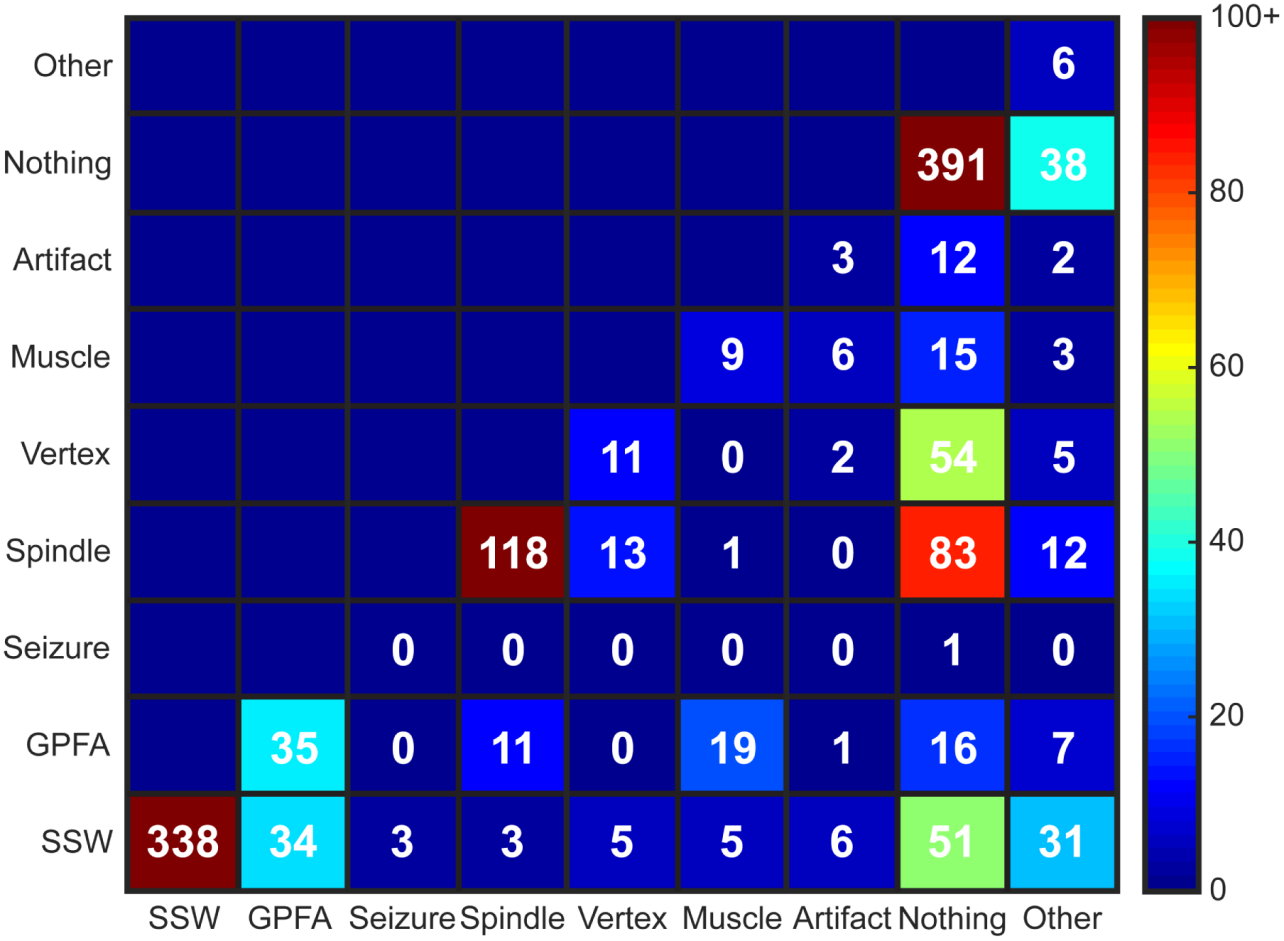
Confusion matrix for waveform classification. Most disagreements were due to one rater labeling the EOI as nothing while the second rater labeled the EOI as something else.

Mismatches in waveform labeling mostly occurred when one rater classified the EOI as nothing (Figure 4). Of the 439 EOIs with a disagreement in waveform labels between the two reviewers, 270 EOIs (61.5%) were labeled as nothing by one reviewer. This happened most frequently for nonepileptiform waveforms; for example, mismatched labels of spindle/nothing occurred 83 times, and labels of vertex/nothing occurred 54 times, accounting for a total of 31.2% of all EOIs with mismatched labels. Of the waveform types typically associated with epilepsy, SSWs had the highest EAR, but 37.0% of all SSWs with mismatched labels occurred when one rater marked nothing. For GPFAs, the mismatches occurred when the second rater chose SSW (38.6%), muscle artifact (21.6%), nothing (18.2%), and sleep spindles (12.5%).

## 4 DISCUSSION

The present study evaluated the rater accuracy and IRR of EEG waveforms in both healthy controls and patients with LGS. The visual classification of 1350 EOIs by three pediatric epileptologists demonstrated favorable interrater agreement in identifying control vs. LGS EEG, using a 15‐second segment of deidentified EEG. The IRR and EAR for waveform type were low, particularly in crucial EEG waveforms such as GPFA and spindles. Mismatched labels for waveforms most frequently occurred when one of the raters labeled the EOI as nothing, suggesting that reviewers had different tolerances for variation in individual waveforms relative to a stereotypical appearance. These results highlight EEG features that can be robustly identified, as well as those with high levels of disagreement between experts. This can guide future work on objectively defining such waveforms and evaluating their utility as biomarkers of diagnosis and treatment response, which will ultimately improve the long‐term outcomes of patients with LGS.

Our results are in line with prior IRR studies on EEG event classification. For example, raters had *κ* =0.43 in marking epileptiform events vs. benign paroxysmal activity.^23^ Another study had clinical experts score 13 262 candidate interictal epileptiform discharges in epilepsy and control subjects that were clustered into groups with similar morphologies.^8^ This study reported an IRR of *κ* =0.487 for the interictal epileptiform activity score, and the authors suggested that the requirement to make binary decisions, rather than assigning probabilities, contributed to the level of disagreement. These findings are consistent with our result that most mismatched labels occurred when one rater classified an EOI as a meaningful EEG waveform while another rater other classified it as nothing.

### 4.1 Automated waveform detection in subjects

In all subjects, the automated process for EEG analysis identified transient waveforms with high power. Of the 794 EOIs with rater agreement, epileptiform waveforms such as SSWs and GPFAs came from fifteen LGS (two control) and eight LGS (zero control) subjects, respectively. In contrast, normal sleep EEG waveforms such as spindles and vertex waves were only seen in three LGS (eighteen controls) and zero LGS (nine controls), respectively. This is consistent with a prior LGS sleep study, which reported an absence of sleep spindles in most LGS subjects.^24^ Sleep spindles and vertex waves may also be difficult to recognize in LGS subjects due to the presence of frequent epileptiform discharges during NREM sleep.^24^, ^25^, ^26^

### 4.2 Rater accuracy for subject identification

The accuracy for classifying subject type was generally good across all three raters, with an average accuracy of 85% across all EOIs. The rater accuracy was similar between controls and LGS subjects in clusters one through six, where raters had a mean classification accuracy of 96.5% for control EOIs and an accuracy of 91.3% for LGS EOIs. However, the rater accuracy in clusters seven through twelve was 95.3% in controls, compared to 64.3% in LGS subjects. This difference was expected, as control subject EEG should only contain normal physiological waveforms, which are unlikely to be mistaken for epileptiform activity. In contrast, LGS subjects have both epileptiform waveforms and normal physiological waveforms, of which the latter are likely to result in mismarking of an EOI as coming from a control subject. Because LGS EOIs more frequently occurred in clusters one through six, rater accuracy for EOIs from LGS subjects decreased as the cluster number increased, with cluster twelve having the lowest accuracy by far (Supplementary Table S3).

### 4.3 Interrater reliability in waveform classification

The combined, blinded rating of both LGS and control subject EOIs in the same study provides an important benchmark for IRR of EEG waveforms. Using an approximately equal mix of physiological and pathological EOIs, let us accurately measure how frequently an EOI might be interpreted as benign or even “nothing” and prevented bias toward choosing labels associated with pathological waveforms. However, this broad mix of EOIs coupled with the use of nine different labels for visual analysis could have contributed to the low values of IRR and EAR for waveform classification. To verify that this was not the case, we recalculated the metrics using three broader categories consisting of: (1) pathological (SSW, GPFA, seizure), (2) physiological (spindle, vertex), and (3) others (muscle, artifact, none, other). The use of fewer labels did not substantially improve IRR (*κ* =0.558 to *κ* =0.628) or the EAR (pathological = 0.724, physiological = 0.447, others = 0.623) (Supplementary Figure S5). The similarities in rater agreement when using broad categories suggest that the disagreement between clinicians is not due to subtle differences between waveform types.

### 4.4 Limitations

There are several limitations to our study related to the EOI detection and subject data. First, the EOI detection was accomplished using the Fz channel, which captures the frontocentral activity typical of spindles, GPFA, and generalized activity, but is insensitive to localized transients occurring elsewhere on the head. Second, the EEG clips were not selected from a specific stage of sleep. Short clips of EEG may not be representative of a subject's brain activity over longer periods of time, as paroxysmal events such as GPFAs and SSWs may appear infrequently or not at all within a 10‐minute time window. However, both limitations would only affect the number of different types of waveforms in the data set and not the raters' ability to classify them.

The LGS subjects used in this study had a wide range of ages and were diverse in terms of seizure and LGS duration and severity of EEG findings. This could have affected our results in a few ways. This could be seen as a strength of the study, as the rater accuracy for labeling EEG segments as LGS or control was quite high, despite the diversity of the LGS cohort. However, it is possible that the accuracy could have been higher in a more homogeneous cohort. With respect to estimation of IRR, drawing individual EEG EOIs from this broad cohort ensured that raters labeled the full spectrum of possible EEG waveforms associated with LGS. Because of this, there were likely “nonstandard” examples of different waveform types, which could have reduced the IRR. However, this represents a realistic scenario, where each EEG requires robust and accurate visual interpretation, regardless of whether its features are typical.

Rater accuracies may have also been affected by the difficulty of classifying individual waveforms compared to conventional EEG visual analysis. The GUI was designed to mimic standard clinical viewing software, but EOI labeling was done using fifteen seconds of isolated EEG, without having the full EEG study for context. We compensated for this by allowing raters to use the entire segment of EEG as context, but this may have influenced some labels by introducing other salient EEG features within the segment. For example, an EOI with short duration (~100 ms) may not initially look significant, but if an epileptiform discharge occurs later in the 15‐second segment, the rater may be more likely to choose a label associated with a pathological waveform. While the use of a deidentified short EEG segment instead of the entire study can reduce bias, future studies may want to evaluate clinically marked waveforms in tandem with automated EOI detections to compare the differences between these two methods. Agreement may be higher in paradigms in which raters assign subject type based on review of full‐length clinical EEG, rather than 15‐second samples. Conversely, in paradigms with diverse patient composition (eg, a cohort including normal controls, patients with LGS, and patients with other forms of epilepsy), the identification of patients with LGS may be more challenging and IRR is likely to be lower.

### 4.5 Future work

Given the stated limitations of this work, there are three questions that could be addressed by subsequent studies, using the same computational analysis and visual marking framework. (1) Is disagreement between raters more likely to occur when there is high uncertainty about the selected label? This could be answered by having raters indicate their level of certainty for each waveform label. If label mismatches occur when raters have high certainty, this could indicate a fundamental disagreement about how standard waveforms should be defined. (2) If raters are provided with greater context for each EEG EOI, does this improve the interrater reliability? For example, raters could assess multiple EOIs from a given subject, with the ability to view all EOIs prior to labeling them. This could be done using all EOIs from a single subject, or it could be subdivided by clusters (eg, label cluster 1 EOIs from subject 1, then cluster 1 EOIs from subject 2, etc.). In theory, this should improve the IRR, particularly for established biomarkers such as epileptiform discharges and the slow spike and wave pattern; results for newer biomarkers such as GPFA may be more varied. (3) What is the reliability of visual labeling in a more realistic scenario in which the patient may have a type of epilepsy other than LGS? Labeling an EOI as “GPFA” is likely to be considerably easier when the rater knows that the subject either has LGS or is a healthy control. Therefore, this study could be repeated using a broader cohort including the various types of epilepsy that are most common in this age group, as well as healthy controls.

Improvements could be made to the computational algorithm used for EEG EOI selection, as well. Future implementations could utilize multiple channels in the time‐frequency analysis, rather than focusing on only channel Fz. Moreover, the use of EEG clips longer than 10 minutes would increase the likelihood of capturing waveforms such as GPFA and SSW in each subject.

## 5 Conclusions

This work is a first step toward understanding the visual interpretation of EEG waveforms relevant to LGS. The IRR reported here should be considered as a baseline level of reliability, given the stringent blinding and randomization used for visual marking. Future studies should address the impact of modifying these factors, such as the amount of EEG context provided and the direct comparison to other forms of epilepsy, to deepen our understanding of this complex topic.

## AUTHOR CONTRIBUTIONS

DH has first authorship of the study and was in charge of the conceptualization, methodology, formal analysis, and the draft manuscript preparations for the study. MR and LD provided clinical expertise and contributed to the data collection and sleep staging. DA, DS, and SH helped supervise the project, provided clinical expertise, performed blinded labeling of the EOIs, and helped review and edit the manuscript. BL is the corresponding author of the study and helped conceptualize, administer, and supervise the project, while reviewing and editing the manuscript.

## CONFLICT OF INTEREST STATEMENT

None of the authors have potential conflicts of interest to be disclosed.

## ETHICAL PUBLICATION STATEMENT

We confirm that we have read the Journal's position on issues involved in ethical publication and affirm that this report is consistent with those guidelines.

## ETHICS APPROVAL

The study was approved by the Institutional Review Boards at the Children's Hospital of Orange County and the University of California Los Angeles, with the requirement for informed consent waived.

## Supporting information


Data S1.

## Data Availability

Anonymized data that support the findings of this study are available from the corresponding author upon request.
