# Supplementary material for: Interrater reliability of interictal EEG waveforms in Lennox–Gastaut Syndrome
Source: Epilepsia Open. 2023 Nov 28;9(1):176–86. doi: 10.1002/epi4.12858 (PMC10839292; doi:10.1002/epi4.12858)
Supplement: Supplementary file 1 — Data S1. [file EPI4-9-176-s001.docx]

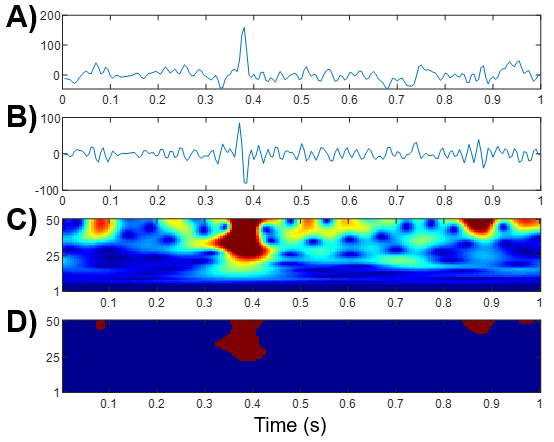


***Supplementary Figure 1****. A simulated spike lasting 20 milliseconds appears as a time-frequency image lasting over 100 milliseconds. (A) A one-second clip of simulated EEG containing a 20-millisecond simulated spike. (B) The pre-whitened version of the signal in Subfigure A. (C) The Time-frequency spectrogram and (D) thresholding at two z-scores (97.7 percentile) show that the simulated spike appears as an island with a duration of approximately 110-milliseconds.*

*
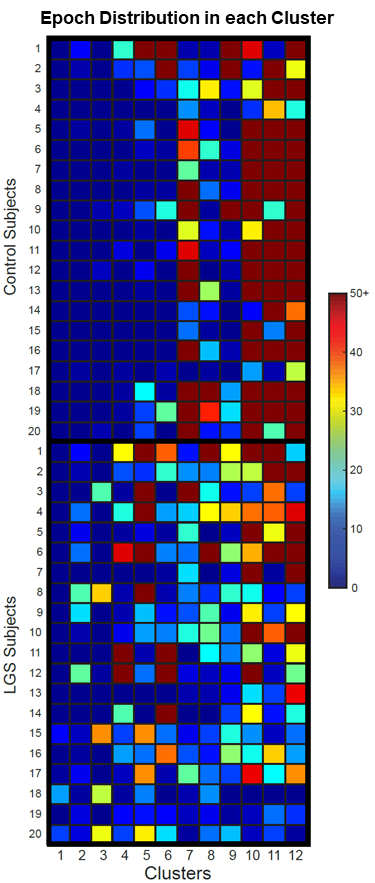
*

***Supplementary Figure 2****. Heatmap showing the number of EOIs in each cluster for controls and LGS subjects, where a warmer color indicates more EOIs. LGS subjects more frequently had EOIs in clusters 1-5, which generally had higher values of the six features. Control subjects had a majority of EOIs in clusters 10-12, which generally had low values of the six features. The maximum color limit was set to 50 for visualization; note that some data points exceeded this value.*

*
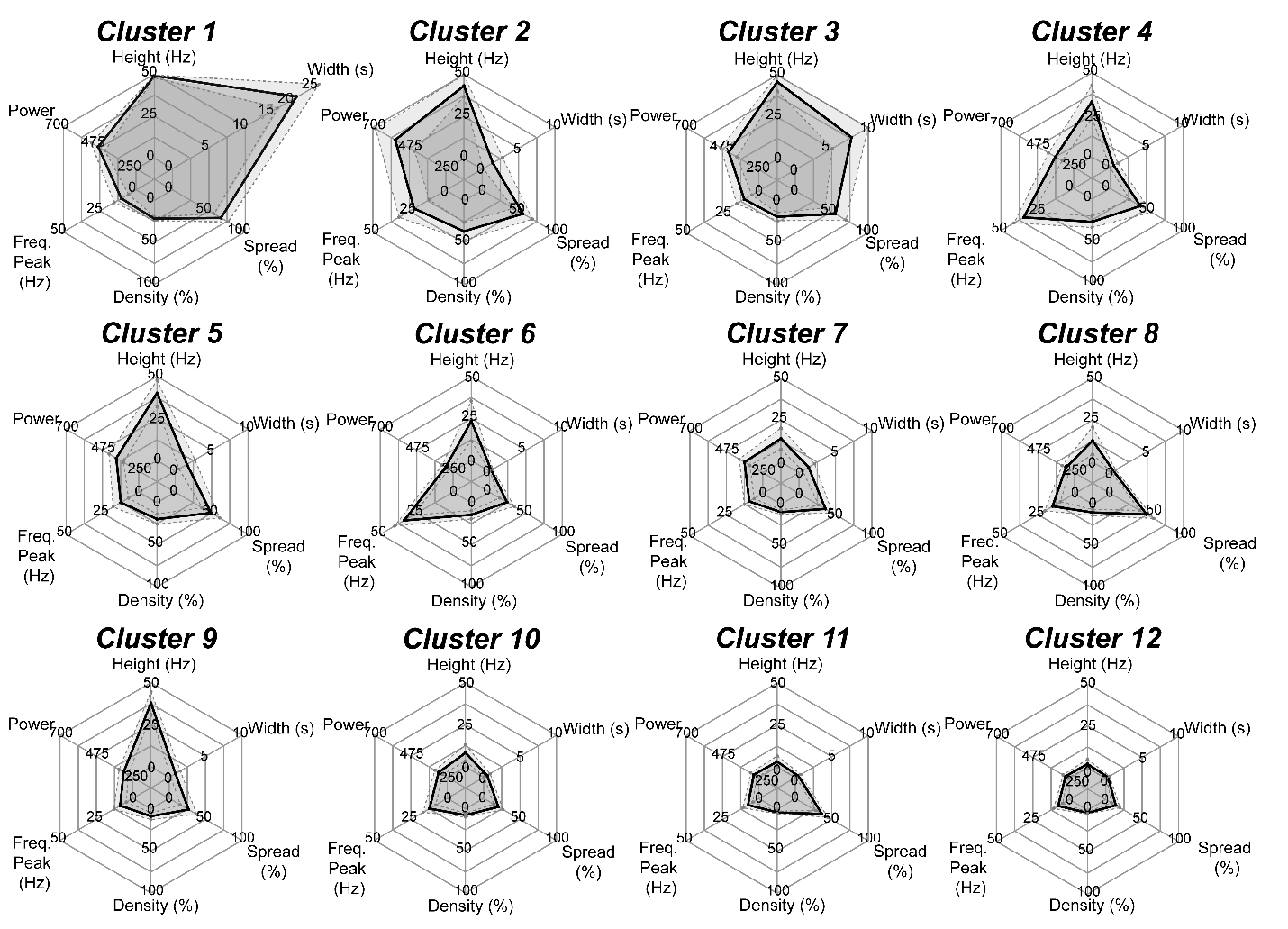
*

***Supplementary Figure 3****. Radar plots showing the mean (black line) and standard deviation*

*(grey dashed line) of the feature values for each of the twelve cluster centroids. Each cluster is*

*characterized by a different combination of the six features.*


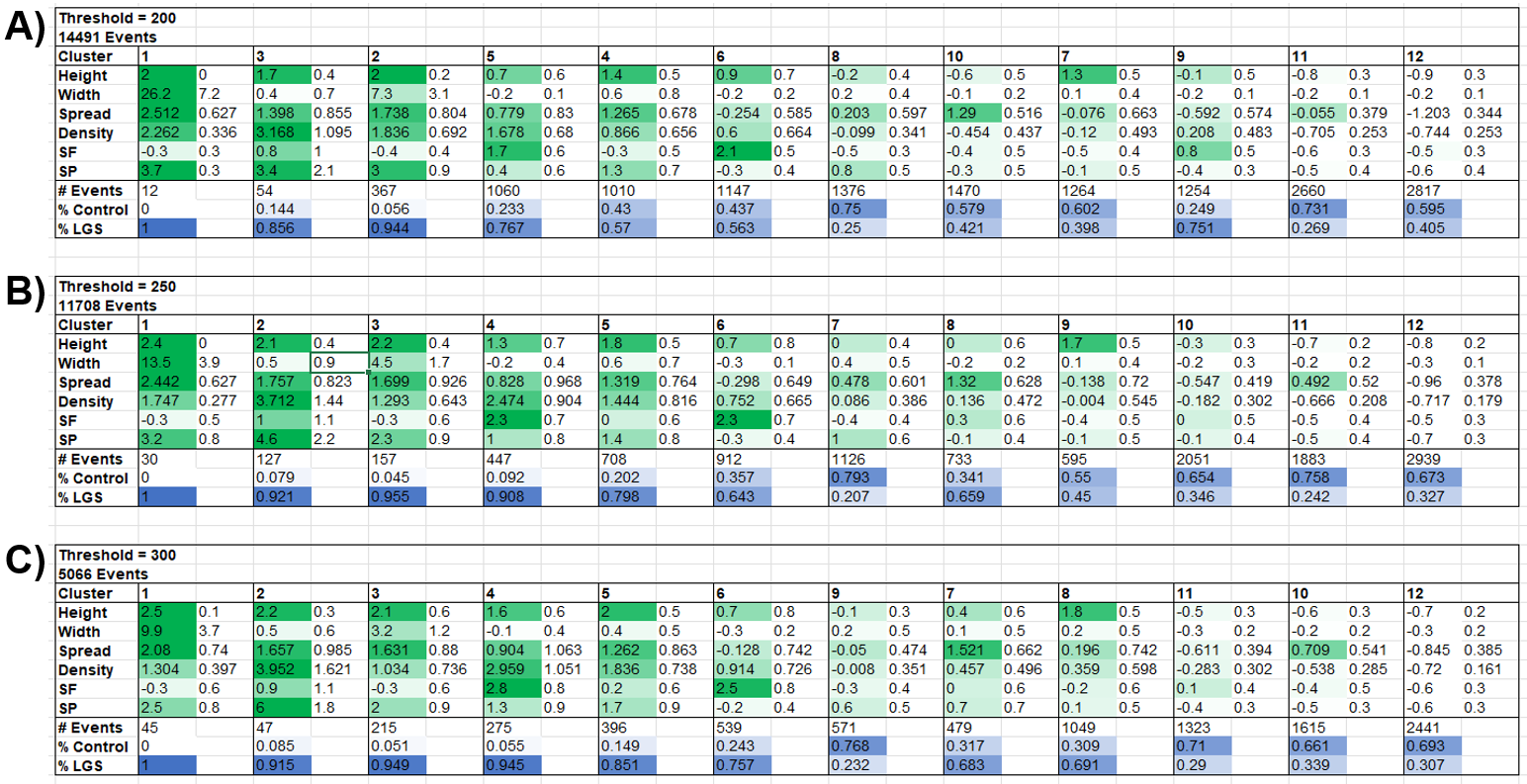


***Supplementary Figure 4****. EOI detection thresholds of (A) 200, (B) 250, and (C) 300 yielded similar cluster centroids with similar ratios of control to LGS EOIs. For each cluster, the mean and standard deviation of the z-score for each feature is shown above, while the percentages of control and LGS EOIs are shown below. The opacity of the green cells indicates the value of the z-score features, with low values appearing white; similarly, the opacity of the blue cells indicates the relative number of control and LGS EOIs. Clusters using a threshold of 250 are sorted in descending order based on the sum of the z-scores; clusters using a threshold of 200 and 300 are sorted to best match the clusters with a threshold of 250.*

*
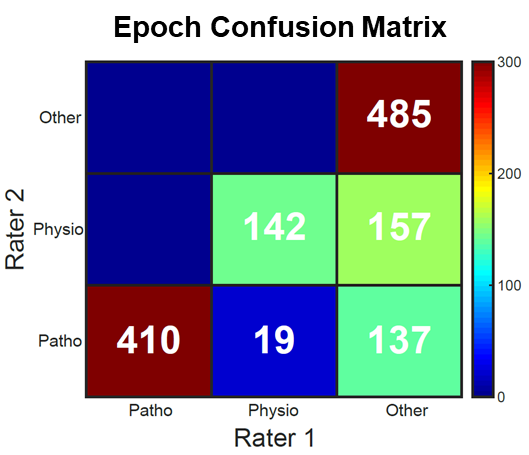
*

***Supplementary Figure 5****. Confusion matrix for waveform type when grouped as pathological (GPFA, SSW, seizures), physiological (spindles and vertex), and other (muscle, artifact, nothing, and other). The interrater reliability of EOIs using broad terms remains inadequate.*

***Supplementary Table 1****. Times between EEG and seizure onset and LGS diagnosis*

| **Subject** | **Sex** | **Age at EEG (years)** | **Age at seizure onset (years)** | **Age at LGS diagnosis (years)** |
| --- | --- | --- | --- | --- |
| **1** | F | 1.4 | 0.7 | 1.1 |
| **2** | F | 18.9 | 0.0 | 16.6 |
| **3** | M | 6.2 | 0.5 | 5.3 |
| **4** | M | 16.5 | 8.3 | 9.7 |
| **5** | M | 9.5 | 5.9 | 9.5 |
| **6** | M | 1.3 | 0.6 | 1.3 |
| **7** | M | 8.0 | 1.0 | 8.0 |
| **8** | F | 7.4 | 0.3 | 7.4 |
| **9** | M | 17.4 | 0.2 | 13.5 |
| **10** | F | 11.5 | 7.7 | 11.4 |
| **11** | F | 1.6 | 1.3 | 1.6 |
| **12** | M | 13.6 | 9.1 | 13.6 |
| **13** | F | 1.6 | 0.5 | 1.6 |
| **14** | M | 8.4 | 0.0 | 7.5 |
| **15** | M | 7.8 | 2.6 | 3.8 |
| **16** | F | 1.0 | 0.0 | 1.3 |
| **17** | M | 7.5 | 4.4 | 7.1 |
| **18** | M | 6.9 | 0.5 | 6.8 |
| **19** | M | 1.5 | 0.9 | 1.5 |
| **20** | M | 2.1 | 0.0 | 1.9 |

***Supplementary Table 2****. Subject type accuracy for visual reviewers A, B, and C.*

| **Rater** | **EOI Count** | | | **Rater Accuracy** | | |
| --- | --- | --- | --- | --- | --- | --- |
|  | **Control** | **LGS** | **All** | **Control** | **LGS** | **All** |
| **A** | 337 | 563 | 900 | 0.956 | 0.778 | 0.844 |
| **B** | 338 | 562 | 900 | 0.979 | 0.767 | 0.847 |
| **C** | 337 | 563 | 900 | 0.926 | 0.821 | 0.860 |

***Supplementary Table 3****. Rater accuracies for subject labels in each cluster and subject group. Clusters are sorted in descending order based on the sum of the z-scores for the six TFI features. Rater accuracy for LGS subjects decreases as the cluster number increases.*

| **Cluster** | **Rater A** | | |  | **Rater B** | | |  | **Rater C** | | |
| --- | --- | --- | --- | --- | --- | --- | --- | --- | --- | --- | --- |
|  | **All** | **Control** | **LGS** |  | **All** | **Control** | **LGS** |  | **All** | **Control** | **LGS** |
| C1 | 1.00 | N/A | 1.00 |  | 1.00 | N/A | 1.00 |  | 1.00 | N/A | 1.00 |
| C2 | 0.94 | 0.88 | 0.94 |  | 0.94 | 1.00 | 0.93 |  | 0.98 | 1.00 | 0.97 |
| C3 | 0.99 | 1.00 | 0.99 |  | 0.98 | 1.00 | 0.97 |  | 0.96 | 1.00 | 0.96 |
| C4 | 0.91 | 1.00 | 0.87 |  | 0.88 | 0.96 | 0.83 |  | 0.93 | 0.96 | 0.91 |
| C5 | 0.89 | 0.86 | 0.91 |  | 0.93 | 0.95 | 0.90 |  | 0.95 | 0.95 | 0.95 |
| C6 | 0.86 | 0.98 | 0.75 |  | 0.85 | 0.98 | 0.73 |  | 0.89 | 0.95 | 0.83 |
| C7 | 0.69 | 0.94 | 0.52 |  | 0.73 | 1.00 | 0.56 |  | 0.71 | 0.94 | 0.54 |
| C8 | 0.91 | 0.92 | 0.90 |  | 0.90 | 0.97 | 0.83 |  | 0.89 | 0.90 | 0.88 |
| C9 | 0.86 | 0.95 | 0.78 |  | 0.89 | 0.98 | 0.80 |  | 0.88 | 0.92 | 0.83 |
| C10 | 0.74 | 1.00 | 0.53 |  | 0.71 | 0.95 | 0.51 |  | 0.80 | 0.89 | 0.71 |
| C11 | 0.89 | 1.00 | 0.78 |  | 0.88 | 1.00 | 0.76 |  | 0.81 | 0.83 | 0.80 |
| C12 | 0.58 | 0.97 | 0.25 |  | 0.61 | 1.00 | 0.26 |  | 0.64 | 1.00 | 0.34 |
